# Reduction of multiplicity of infections but no change in *msp2* genetic diversity in *P.falciparum* isolates from Congolese children after introduction of artemisin-combination therapies

**DOI:** 10.1186/1475-2875-11-S1-P70

**Published:** 2012-10-15

**Authors:** Rod Ibara-Okabande, Felix Koukouikila-Koussounda, Mathieu Ndounga, Jeannhey Vouvoungui, Vladimir Malonga, Prisca Nadine Casimiro, Jean Rosaire Ibara, Anissa Sidibe, Francine Ntoumi

**Affiliations:** 1 Congolese Foundation for Medical Research, Brazzaville, Republic of Congo; 2 Centre de Recherche sur les Ressources Végétales, Brazzaville, Republic of Congo; 3 Centre d’Etudes sur les Resources Vegetales, Brazzaville, Republic of the Congo; 4 Université Marien Ngouabi, Republic of Congo

## Background

In this first study conducted after the introduction of artemisin-combination therapies, we investigated the genetic diversity of *P. falciparum* isolates from children aged 1-9 years enrolled and followed up for one year to investigate clinical malaria cases. In addition, the msp2 profiles of *P. falciparum* isolates collected from successive malaria episodes in ten children who had four or more clinical episodes during the follow up were characterized. Three hundred and thirteen children residing in Southern part of Brazzaville participated in this study. Blood samples were obtained from all children at enrollment and checked for *P. falciparum* infection. Based on the one year follow-up data, two clinical groups were considered according to the number of malaria episodes presented over the follow up period: “protected” (children who did not experience any episode) and “unprotected” (those who experienced more that two episodes). Therefore, the msp2 genetic diversity of *P. falciparum* isolates collected at enrollment in the two groups was characterized by allele-specific nested PCR and compared. The msp2 profiles of *P. falciparum* isolates collected from successive malaria episodes was also characterized by allele-specific nested PCR. We found 43% FC27 and 57% 3D7 in protected vs 56% FC27 and 44% 3D7 in isolates from unprotected children. Seven and two alleles belonging to the FC27, and six and three alleles belonging to 3D7 families were distinguished in isolates from protected and unprotected children respectively. The mean MOI values at inclusion for the msp2 locus were 1.29 and 1.43 for protected and unprotected children respectively. 43 isolates were obtained from the ten children who had four or more clinical episodes during the follow up. A total of 63 alleles or fragments corresponding to 56% (36/63) FC27 and 44% (27/63) 3D7 were detected. The variant 400bp of FC27 was the most prevalent. 46% (20/43), 42% (18/43), 2% (1/43) and 2% (1/43) of isolates were found to have 1, 2, 3 and 4 parasite genotypes respectively and the mean MOI was 1.78.

## Conclusion

This study shows that the introduction of ACTs in the Republic of Congo has reduced the multiplicity of infection but not the genetic diversity of *P.falciparum* isolates from children living in Southern districts of Brazzaville.

